# Utilization of a Wheat660K SNP array-derived high-density genetic map for high-resolution mapping of a major QTL for kernel number

**DOI:** 10.1038/s41598-017-04028-6

**Published:** 2017-06-19

**Authors:** Fa Cui, Na Zhang, Xiao-li Fan, Wei Zhang, Chun-hua Zhao, Li-juan Yang, Rui-qing Pan, Mei Chen, Jie Han, Xue-qiang Zhao, Jun Ji, Yi-ping Tong, Hong-xia Zhang, Ji-zeng Jia, Guang-yao Zhao, Jun-ming Li

**Affiliations:** 1 Center for Agricultural Resources Research, Institute of Genetics and Developmental Biology, Chinese Academy of Sciences, Shijiazhuang, 050022 China; 2 Genetic Improvement Centre of Agricultural and Forest Crops, College of Agriculture, Ludong Unversity, Yan’tai, 264025 China; 3 Chengdu Institute of Biology, Chinese Academy of Sciences, Chengdu, 610041 China; 4 Xinxiang Academy of Agricultural Sciences, Xinxiang, 453000 China; 5 Institute of Crop Science, Chinese Academy of Agricultural Sciences, Beijing, 100081 China; 6 University of Chinese Academy of Sciences, Beijing, 10049 China; 7 State Key Laboratory of Plant Cell and Chromosomal Engineering, Chinese Academy of Sciences, Beijing, 100101 China

## Abstract

In crop plants, a high-density genetic linkage map is essential for both genetic and genomic researches. The complexity and the large size of wheat genome have hampered the acquisition of a high-resolution genetic map. In this study, we report a high-density genetic map based on an individual mapping population using the Affymetrix Wheat660K single-nucleotide polymorphism (SNP) array as a probe in hexaploid wheat. The resultant genetic map consisted of 119 566 loci spanning 4424.4 cM, and 119 001 of those loci were SNP markers. This genetic map showed good collinearity with the 90 K and 820 K consensus genetic maps and was also in accordance with the recently released wheat whole genome assembly. The high-density wheat genetic map will provide a major resource for future genetic and genomic research in wheat. Moreover, a comparative genomics analysis among gramineous plant genomes was conducted based on the high-density wheat genetic map, providing an overview of the structural relationships among theses gramineous plant genomes. A major stable quantitative trait locus (QTL) for kernel number per spike was characterized, providing a solid foundation for the future high-resolution mapping and map-based cloning of the targeted QTL.

## Introduction

High-density genetic linkage maps are essential for genetic and genomic research in crops^1–4^. Molecular breeding is more effective if the molecular map is dense to provide more choices in the quality and type of markers and to increase the probability of detecting polymorphic markers in important chromosomal intervals. In wheat, the large genome size (17 gigabases), hexaploid nature (AABBDD), high percentage of repetitive regions and low level of polymorphism have complicated the acquisition of high-resolution genetic maps by molecular markers^1–4^. To date, several kinds of molecular markers, including restriction fragment length polymorphism (RFLP)^5, 6^, amplified fragment length polymorphism (AFLP)^7^, simple sequence repeats (SSRs)^8, 9^, and diversity array technology (DArT)^3, 4, 10, 11^ have been used to construct genetic linkage maps in wheat. Information regarding wheat molecular markers and genetic maps is available in some datasets such as GrainGenes 2.0 (https://wheat.pw.usda.gov/GG3/), URGI (https://urgi.versailles.inra.fr/), etc. Most of these markers are mapped on the telomeric regions, and there is very limited map resolution in proximal part of the chromosomes^3^. Therefore, the density and coverage of the current genetic maps are less than satisfactory.

Single-nucleotide polymorphisms (SNPs) are the most abundant type of molecular marker. Accurate and reliable methods have been developed to perform high-throughput genotyping based on SNPs^12^. With the development of new sequencing technologies, increasing numbers of SNPs have been discovered in wheat^1, 2, 13–15^. Cavanagh *et al*.^12^ released a hexaploid wheat consensus genetic map with 7504 SNP markers from the Wheat9K SNP array using a combination of seven mapping populations. Wang *et al*.^16^ mapped 46977 SNPs from the Wheat90K array to the hexaploid wheat genetic map using a combination of eight mapping populations. Using both Wheat9K and Wheat90K arrays, Maccaferri *et al*.^17^ released a high-density tetraploid wheat consensus genetic map with 30144 markers (including 26626 SNPs and 791 SSRs) by integrating 13 data sets from independent biparental mapping populations. Recently, Winfield *et al*.^18^ documented a hexaploid wheat consensus map with 56 505 SNP markers from the Wheat820K array, spanning 3739 cM in length, using three independent biparental populations. However, although a high-density hexaploid wheat genetic map (>100 000 markers) based on an individual biparental mapping population would be valuable for further genetic research, such as high-resolution mapping and map-based cloning of a targeted major quantitative trait locus (QTL), no such map has been released.

A new Whole Genome Shotgun (WGS) assembly of the Chinese Spring (CS) reference wheat genome is now available (http://plants.ensembl.org/index.html; https://urgi.versailles.inra.fr/download/iwgsc/IWGSC-WGA_Sequences/). However, genetic and genomic studies in wheat continue to lag behind the research in other members of the grass family (*Gramineae*), such as rice and maize. The gradual enrichment of SNP markers and the sequences released for CS (https://urgi.versailles.inra.fr/download/iwgsc/IWGSC-WGA_Sequences/), *Triticum urartu* ^2^ and *Aegilops tauschii* ^1^ have facilitated comparative genomic analysis in wheat. Comparative genomic analysis with species whose genomes have been well characterized has been used as an effective method for the construction of high-resolution genetic linkage maps of target wheat genes and for the prediction of candidate genes in regions of interest. For instance, the construction of high-density genetic maps has facilitated the mapping of the gene grain protein content-B1 (*Gpc-B1*)^19^, the yellow rust resistance gene *Yr36* ^20^ and the powdery mildew resistance gene *Pm41* ^21^. Comparative genomics studies have also furthered the understanding of the basic processes of genome evolution and the transfer of information from model species to related organisms and facilitated the cross-referencing of various types of information, such as QTLs, mutants, and gene expression^22, 23^. These correlations and integrations will take full advantage of the collective intellectual contributions from scientists across many disciplines^22^.

Wheat660K, the Affymetrix® Axiom® Wheat660, was designed by the Chinese Academy of Agricultural Sciences and synthesized by Affymetrix. This Wheat660K SNP array is genome-specific with high density and is highly efficient with a wide range of potential applications (http://wheat.pw.usda.gov/ggpages/topics/Wheat660_SNP_array_developed_by_CAAS.pdf). However, genetic position in relation to Wheat660K SNPs has not yet been documented. In this work, for the first time, we report a high-density map for wheat constructed from this Wheat660K SNP Array. Based on SNP flanking sequences, we assigned SNPs to the genome assembly of *T. aestivum* cv. Chinese Spring (CS) (https://urgi.versailles.inra.fr/download/iwgsc/IWGSC-WGA_Sequences/). We also compared our high-density genetic map with the consensus genetic maps of Wheat90K and Wheat820K based on the common contigs assembled in the chromosome survey sequencing (CSS) project. Comparative genomic analyses based on the mapped SNP flanking sequences and the corresponding contig sequences were also performed with the genomes of *Brachypodium distachyon*, *Oryza sativa*, *Zea mays*, and *Sorghum bicolor*. Using this mapping resource along with the phenotypic data, we identified important QTLs for yield-related trait. A major stable QTL for kernel number was identified and then characterized in detail based on the high-density genetic map and comparative genomic analysis.

## Results

### SNP scores in the 265 accessions

An F_8:9_ recombinant inbred line (RIL) population including 188 lines derived from a cross between Kenong 9204 (KN9204) and Jing 411 (J411) (denoted as KJ-RILs), 65 KN9204-derived advanced lines/authorized varieties, three parental lines of KN9204, three control varieties from the Winter Wheat Performance Trial of the Northern Huang-Huai Regional Nursery of China, and Chinese Spring (CS) were genotyped using the 630517 SNPs on the Wheat660K SNP array as probes. The sample call rates ranged from 18.6% to 100.0%, with an average of 98.9% for the 265 accessions (data not shown). The scores for the probes were classified into one of the following six categories according to the cluster patterns produced by the Affymetrix software (Table S1): (i) Poly High Resolution (PHR) (188040; 29.8%); (ii) No Minor Homozygote (NMH) (133246; 21.1%); (iii) Off-Target Variant (OTV) (18471; 3.0%); (iv) Mono High Resolution (MHR) (163308; 25.9%); (v) Call Rate Below Threshold (CRBT) (22635; 3.6%); and (vi) Other (91425, 14.5%). Only the first three groups (PHR, NMH and OTV) were considered useful, and they accounted for 53.9% of the Wheat660K SNP array probes.

Of the 339757 functional SNPs, 136973 (40.3%) were polymorphic between KN9204 and J411. Of these, 8407 had more than 10% missing data in the 188 KJ-RILs, and were removed from the linkage analysis. Among the remaining 128566 SNPs, 90567 (70.4%) were transitions, and 37999 (29.6%) were transversions. The 128566 functional SNPs and the previously reported 591 loci^3^ were used for linkage analysis and map construction. The 129157 markers fell into 5175 bins, and to create a chromosome frame, only one marker was selected as a representative from each bin.

### Overview of the high-density wheat genetic map

**Figure 1:**
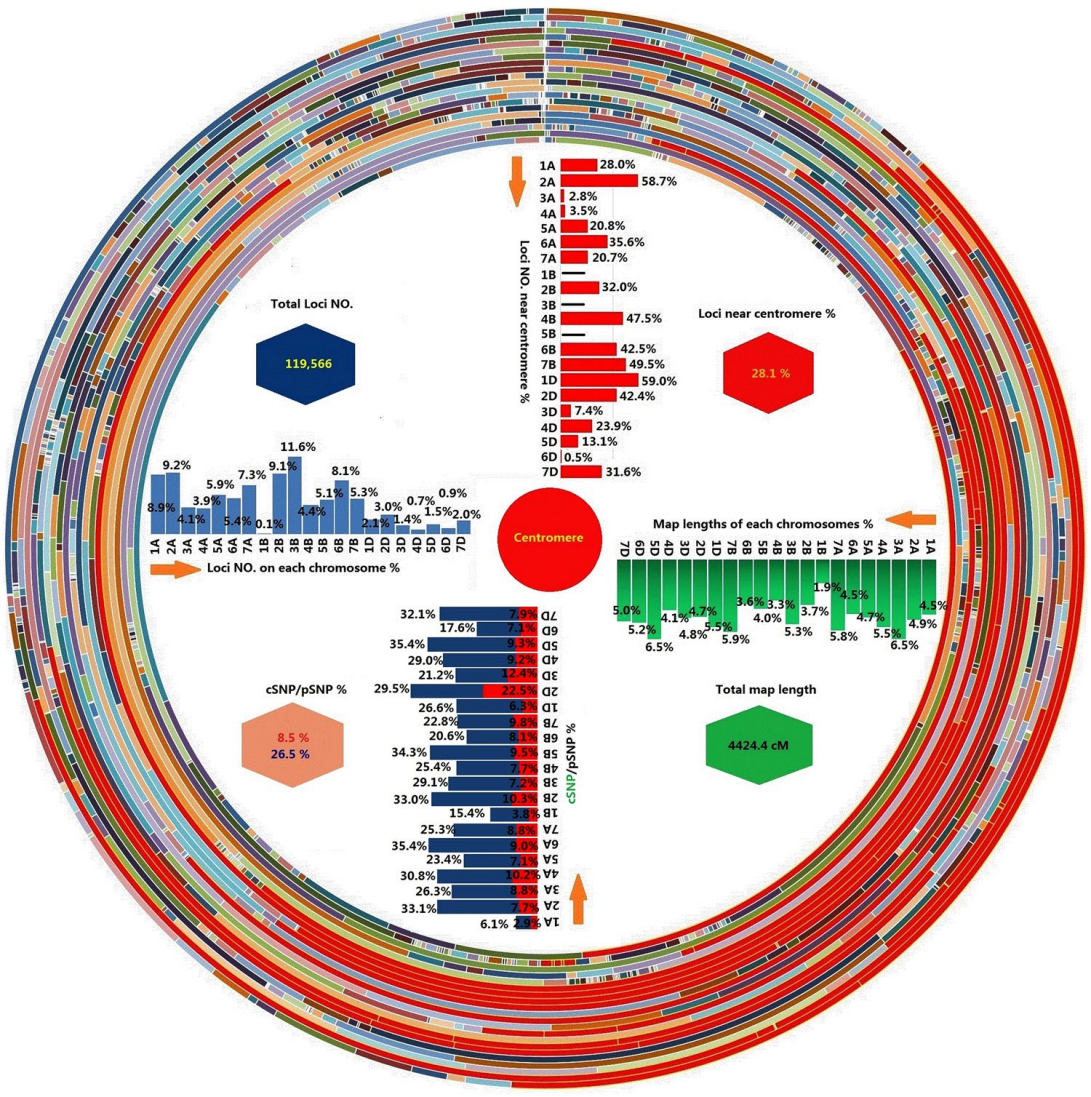
Distribution of the 119 566 loci on the 21 wheat chromosomes. The 21 circles indicate the 21 wheat chromosomes, with 7D to 1D, 7B to 1B, and 7A to 1A from inside to outside, respectively. Each chromosome was evenly divided into 100 segments based on the map length, which are shown with different colours. The arc lengths indicate the percentage of markers on each segment (The total number of markers on the corresponding chromosome divided by the number of markers on the segments). The arc in red colour indicates the chromosomal bins near the centromere. The upper left histogram in the circle indicates the distribution of markers on each chromosome; the upper right histogram in the circle indicates the distribution of markers near the centromere on each chromosome; the bottom right histogram in the circle indicates the distribution of map length on each chromosome; the bottom left histogram in the circle indicates the distribution of markers in/near the coding sequences.

**Figure 2:**
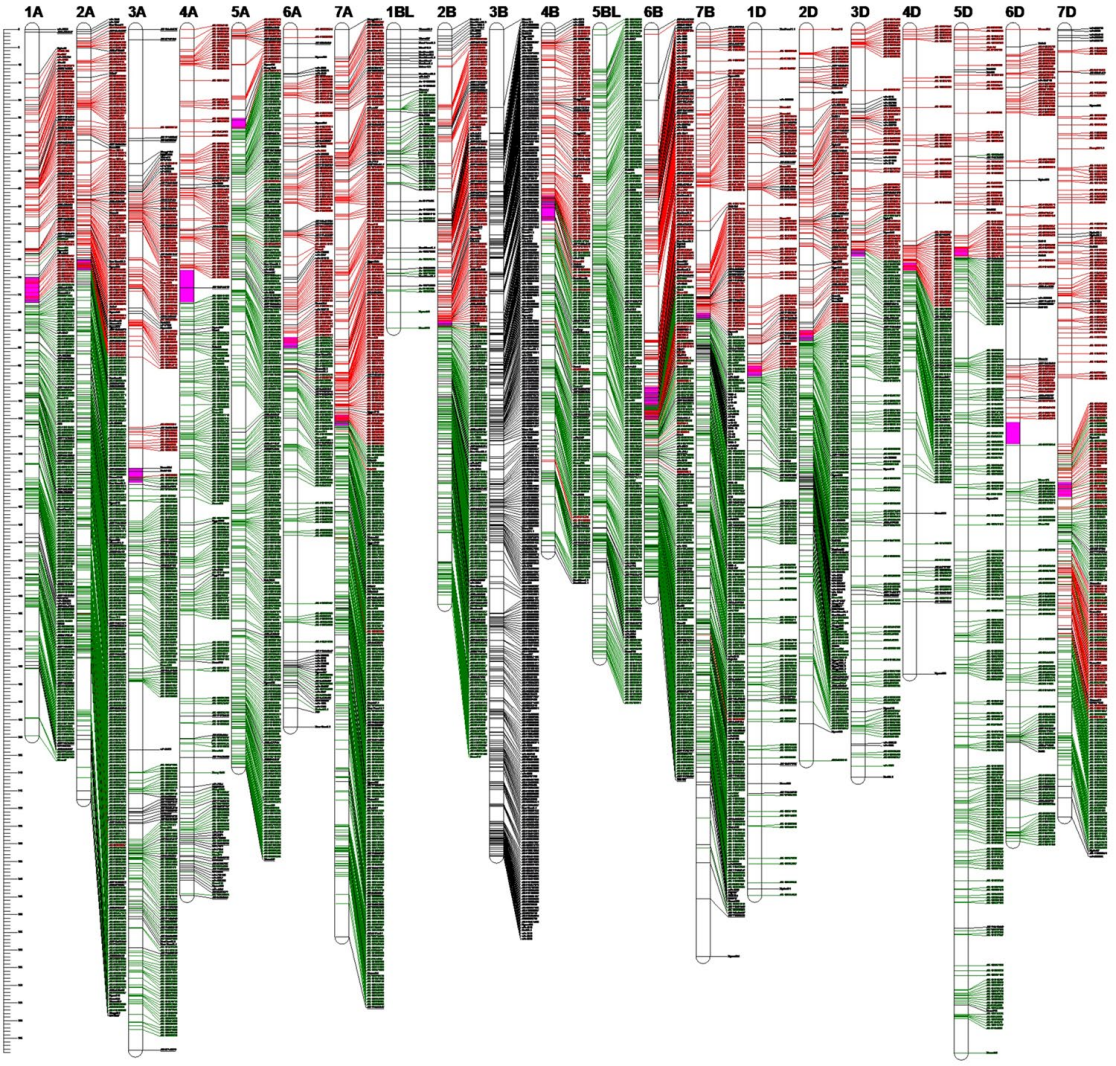
The high-density genetic map of wheat developed using an RIL population derived from the cross of cultivars KN9204 and J411. For the redundant loci that showed co-segregation in the 188 KJ-RILs, only one unique informative marker is shown in this figure. The approximate positions of the centromeres are indicated by *pink*. Short arms are at the *top*. The positions of the marker loci are indicated using a ruler on the left side of this figure. The names of the marker loci are listed to the right of the corresponding chromosomes. Loci in *red* were best hits to Chinese Spring (CS) contigs of the short arm of the corresponding chromosomes. Loci in *green* were best hits to CS contigs of the long arm of the corresponding chromosomes. Loci in *black* were unknown. Contigs from chromosome 3B were not separated into short/long arm bins, as individual arm datasets were not generated for this chromosome in the Chromosome Survey Sequence (CSS) project.

The 5175 bin markers were used for linkage analysis based on their scores in the 188 KJ-RILs. In total, 4959 bin markers were mapped to the wheat genetic map. The co-segregated markers (redundant markers) were then added to the high-density genetic map based on information of bin serial number and the genetic information of the corresponding bin markers. A high-density genetic map with 119566 loci spanning 4424.4 cM was constructed (Table S2). Of these loci, 119001 were SNP markers derived from the Wheat660K SNP array, and the remaining 565 markers were reported previously by Cui *et al*.^3^. Of the 119001 SNPs, 83953 (70.5%) were transitions, and 35048 (29.5%) were transversions (data not shown). Most markers were mapped to the B (44.6%) and A genomes (43.7%), and only 11.7% markers were mapped to the D genome. For the map lengths, the A, B, and D genomes covered 36.4%, 27.7%, and 35.8% of the total map length, respectively. The chromosome sizes ranged from 84.4 cM (chromosome 1BL) to 289.1 cM (chromosome 5D), averaging 210.7 cM per chromosome. The number of markers on each chromosome ranged from 78 (chromosome 1BL) to 13898 (chromosome 3B), with a mean of 5693.6 loci per chromosome. Due to the 1BL/1RS translocation of KN9204, the 1RS- or 1BS-specific markers not only showed co-segregation but also exhibited distorted segregation in the KJ-RILs as shown by Cui *et al*.^3^. These markers were excluded from the linkage analysis and map construction, which reduced the genetic maps for analysis to 1BL only. In addition, no markers on chromosome 5BS were polymorphic between KN9204 and J411, resulting in the release of the 5BL genetic map only. Of the 119566 loci, 33598 (28.1%) were distributed on chromosomal regions near the centromeres. Marker density per genetic distance unit peaked at the centromeric regions, possibly due to a combination of low recombination rate in the centromeric regions and even gene distribution along the chromosomes (Fig. 1). The 4 959 bin markers are shown in the genetic map (Fig. 2). The following mapping-bin sets were observed: approximately 3.7% and 3.2% of the markers were unique for genomes A and B, respectively, and approximately 9.6% of the markers for the D genome showed unique segregation patterns.

Considering the unique markers (the 4 959 bin markers), the highest marker saturation was found in genome A (39.3%), followed by genomes B (33.6%) and D (27.1%). The average distance between adjacent bin markers ranged from 0.6 cM for 6B to 1.5 cM for 4D, with an average of 0.9 cM per marker pair. Gaps greater than 20.0 cM but less than 30.0 cM were present in chromosomes 3 A (24.9 cM), 3B (21.9 cM) and 4D (20.4 cM); gaps greater than 10 cM but less than 20.0 cM were present in chromosomes 1 A (13.9 cM, 11.7 cM), 2 A (14.6 cM), 2B (10.6 cM), 2D (10.5 cM), 3 A (19.9 cM, 19.2 cM), 3D (12.1 cM), 4D (11.5 cM, 11.2 cM, 11.0 cM, 11.0 cM), 5 A (12.1 cM), 5D (13.2 cM), 6 A (19.2 cM), 6B (13.8 cM, 13.2 cM, 12.7 cM), 6D (14.6 cM, 13.6 cM, 10.9 cM, 10.7 cM), 7B (16.3 cM, 10.1 cM), and 7D (18.8 cM) (Fig. 2).

### Comparative genomic analysis

**Figure 3:**
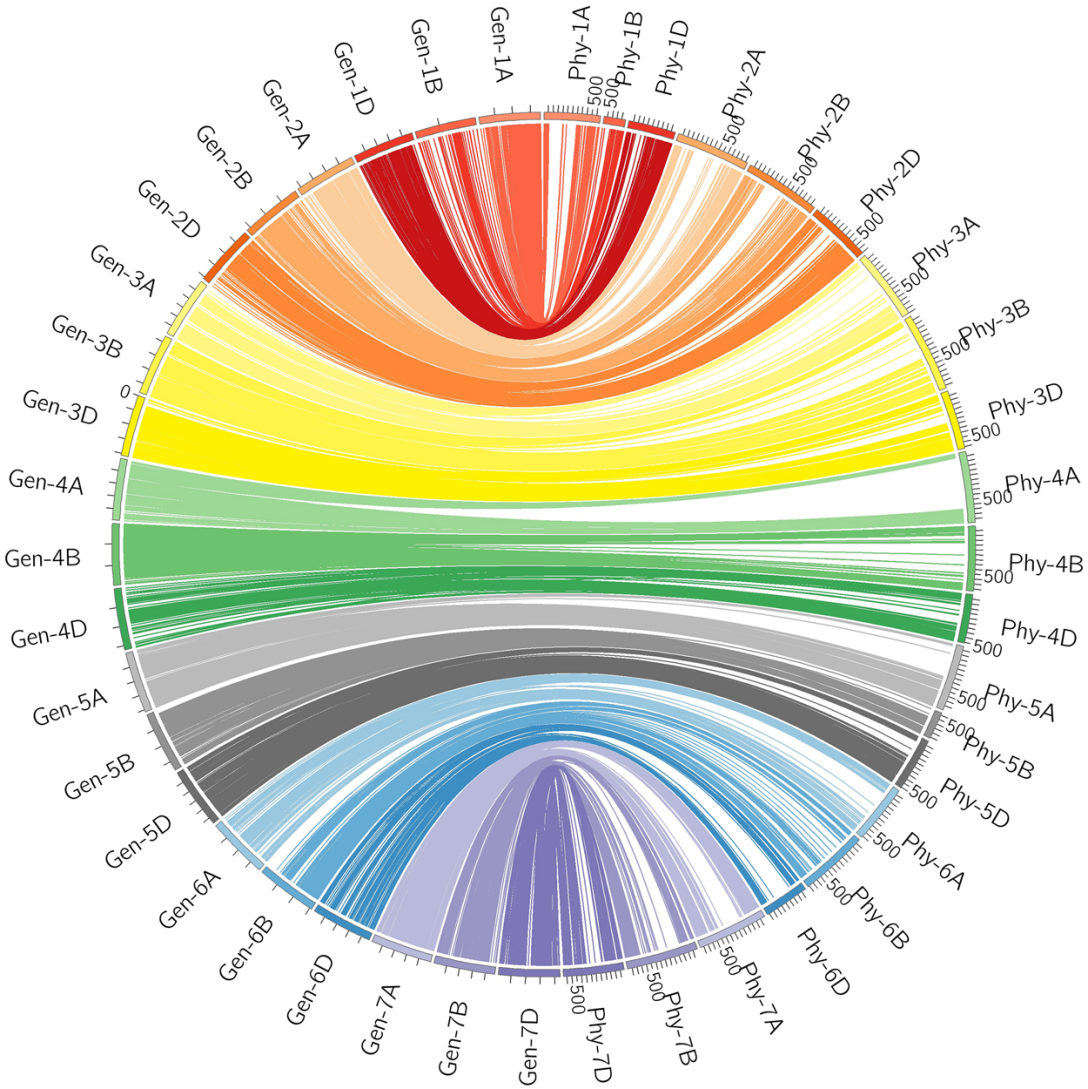
Schematic representation of the syntenic relationships between any one marker in wheat genetic and physical maps. Gen-1A to Gen-7D represent the 21 wheat chromosomal genetic maps released in this paper; Phy-1A to Phy-7D represent the 21 wheat chromosomal physical maps, which were constructed by assigning 116 261 SNPs to the wheat genome assembly using SNP flanking sequences as the query. For the redundant loci that showed co-segregation in the 188 KJ-RILs, only one unique informative marker is shown in this figure.

Of the 119566 loci, 118998 (99.5%) were best hits to 57036 CSS contigs, with 2.1 polymorphic markers per contig. In total, 93.0% contigs had coincident physical and genetic positions, 4.6% were mapped to homoeologous chromosomes such as 1 A in physical position and 1B in the KJ-RIL genetic map, and 2.4% were disordered (Fig. 2; Figure S1; Table S3). Based on the SNP flanking sequences, we assigned 116 261 SNPs to the recently released wheat genome assembly. SNP order in the present genetic map was in good agreement with that in the wheat genome assembly, with the exception of chromosome 7DL, in which a segment inversion was identified (Fig. 3; Figure S2; Table S3).

An overview (from the wheat genetic map perspective) of the relationships between the five grass family genomes at the resolution of the genetic map in centimorgans is provided in Figures S3 and S4. There were 113288 markers that corresponded to the CDS of *Brachypodium* (65357 markers) (Table S4; Figure S3a, S4a), rice (58825 markers) (Table S4; Figures S3b, S4b), maize (53745 markers) (Table S4; Figures S3c, S4c), and sorghum (59994 markers) (Table S4; Figures S3d, S4d). In general, chromosomes belonging to the same homoeologous groups showed correspondence with the same grass chromosomes, but some differences were observed. Large and especially small synteny blocks across wheat and grass family chromosomes were observed, indicating the complexity of the wheat genome among the grass family genomes. The structural relationships among the genomes indicate that for some individual wheat chromosomes, there is a preponderance of corresponding grass genes from one or two certain linkage groups. For example, wheat chromosomes 2 A/2B/2D corresponded to Bd5, wheat chromosomes 3 A/3D corresponded to Bd2 and Bd3, wheat chromosomes 4 A/4B/4D corresponded to Os3, and wheat chromosomes 7 A/7B/7D corresponded to Sb10. However, for most synteny blocks, the chromosomes were more fragmented and scattered, with a high frequency of breakdown.

To specify the coding-region SNPs (cSNPs), perigenic SNPs (pSNPs), and intergenic SNPs (iSNPs) among the SNP markers mapped in the KJ-RIL genetic map, a BLASTX search was performed against the CDS of *T. aestivum*, using SNP flanking sequences and the corresponding contig sequences as queries. Using SNP flanking sequences as the query, 8.9% (10104 SNPs) were best hits to the CDSs of the *T. aestivum*, and these were considered to be the cSNPs. When using the contig sequences where the markers were best hits to as the query, approximately 36.8% (41689 SNPs) were best hits for the CDSs of the *T. aestivum*, indicating that 27.9% (36.8–8.9%) of SNPs were pSNPs. The remaining 63.2% are likely iSNPs (Table S5).

### QTLs for KNPS and the prediction of candidate genes

**Figure 4:**
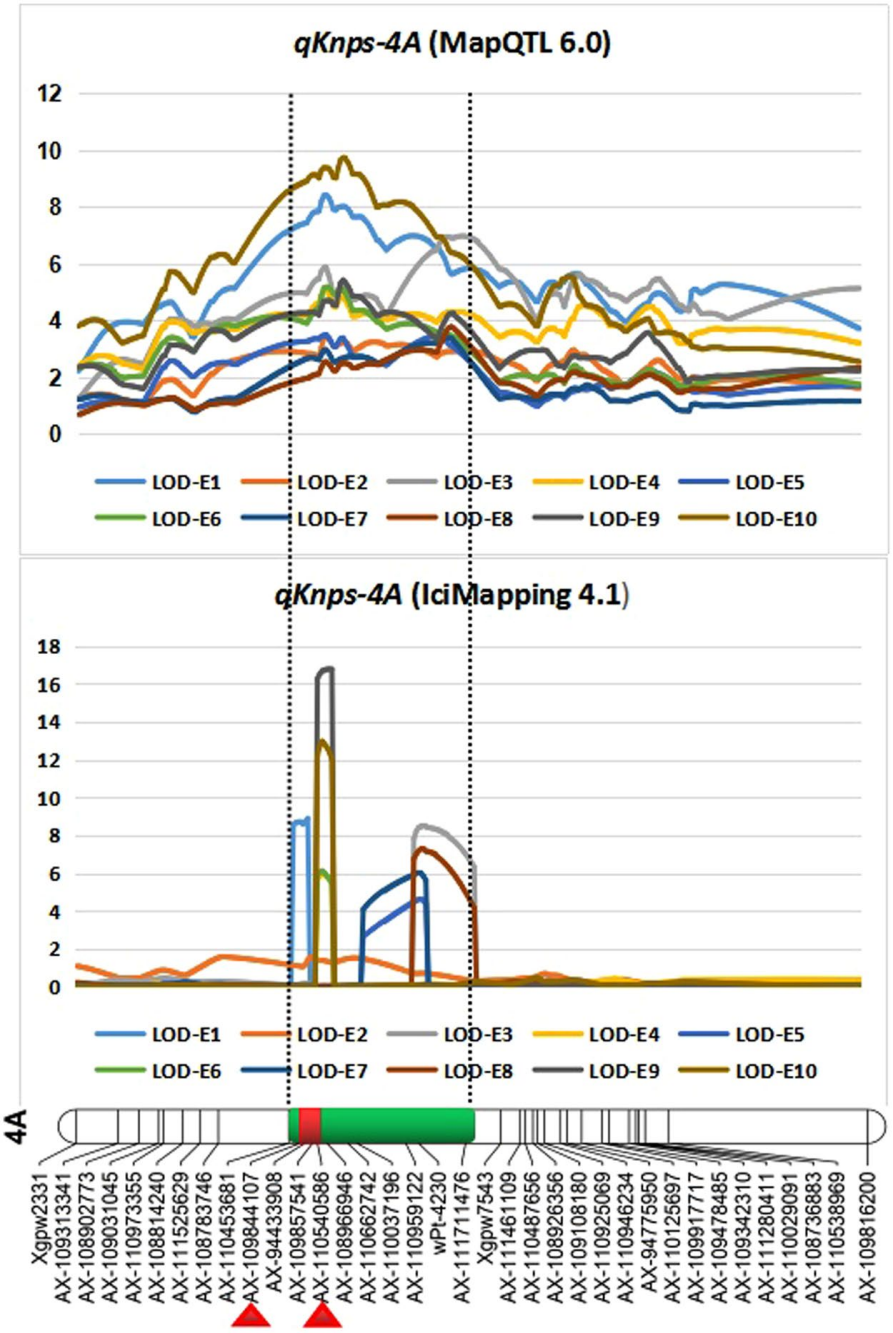
Overlapping confidence intervals of QTLs for kernel number per spike (KNPS) based on MapQTL 6.0, IciMapping 4.1 and QTLNetwork 2.0. The segments in *red* are the overlapping confidence intervals of *qKnps-4A*, and the overlapping flanking markers of *qKnps-4A* are indicated by triangles in *red*, which were detected by QTLNetwork 2.0 based on the combined 10 environmental phenotypic values.

Using the mapping resource along with the phenotypic data we identified important QTLs for yield-related trait. A major stable QTL for kernel number per spike (KNPS; *qKnps-4A*) was verified in 10 environments by using MapQTL 6.0, IciMapping 4.1, and QTLNetwork 2.0 (Table 1; Fig. 4; Figures S5, S6). *qKnps-4A* contributed to 8.0–21.2% of the KNPS phenotypic variation in the 188 KJ-RILs, with alleles from J411 increasing kernels per spike by 1.2–2.4. The QTL peaks of *qKnps-4A* were in 149.8–155.0 cM, 149.7–154.5 cM, and 149.8 cM, as detected by MapQTL 6.0, IciMapping 4.1, and QTLNetwork 2.0, respectively, with *Ax-109844107–Ax-110540586* in 149.6–150.3 cM in the overlapping confidence intervals. Thus, we predicted that the candidate genes for *qKnps-4A* might be within the overlapping confidence intervals of 0.7 cM. Based on the genome assembly of *T. aestivum* cv. CS (https://urgi.versailles.inra.fr/download/iwgsc/IWGSC-WGA_Sequences/), the overlapping confidence intervals of *Ax-109844107–Ax-110540586* spanned 3.23 Mb (4 A:680398739*–*4 A:683638403) in physical position (Figure S7). This region harbours 65 predicted genes in wheat (Figure S8), which might include the candidate gene for *qKnps-4A*.

**Table 1:** *qKnps-4A* as detected by MapQTL 6.0, IciMapping 4.1, and QTLNetwork 2.0.

Software	LOD value	Position (cM)	Additive effect	PVE %
MapQTL 6.0	3.4 to 9.7	149.8 to 155.0	−1.5 to −2.4	8.0 to 21.2
IciMapping 4.1	2.8 to 16.8	149.7 to 154.5	−1.2 to −2.4	9.5 to 20.3
QTLNetwork 2.0	*P* value: 0.000000	149.8	−1.9	10.9

## Discussion

The high-density SNP map developed in the present study first documented the genetic positions of 119 001 SNPs from the Wheat660K SNP array. Based on the SNP flanking sequences, we assigned 118 785 SNPs to 56 904 CSS-assembled contigs (Table S3). The physical positions of the corresponding CSS-assembled contigs could be used to validate genetic position (chromosome and chromosomal arms assignment). As shown in Fig. 2 and Table S3, the physical and genetic positions of these mapped markers were generally in agreement.

**Figure 5:**
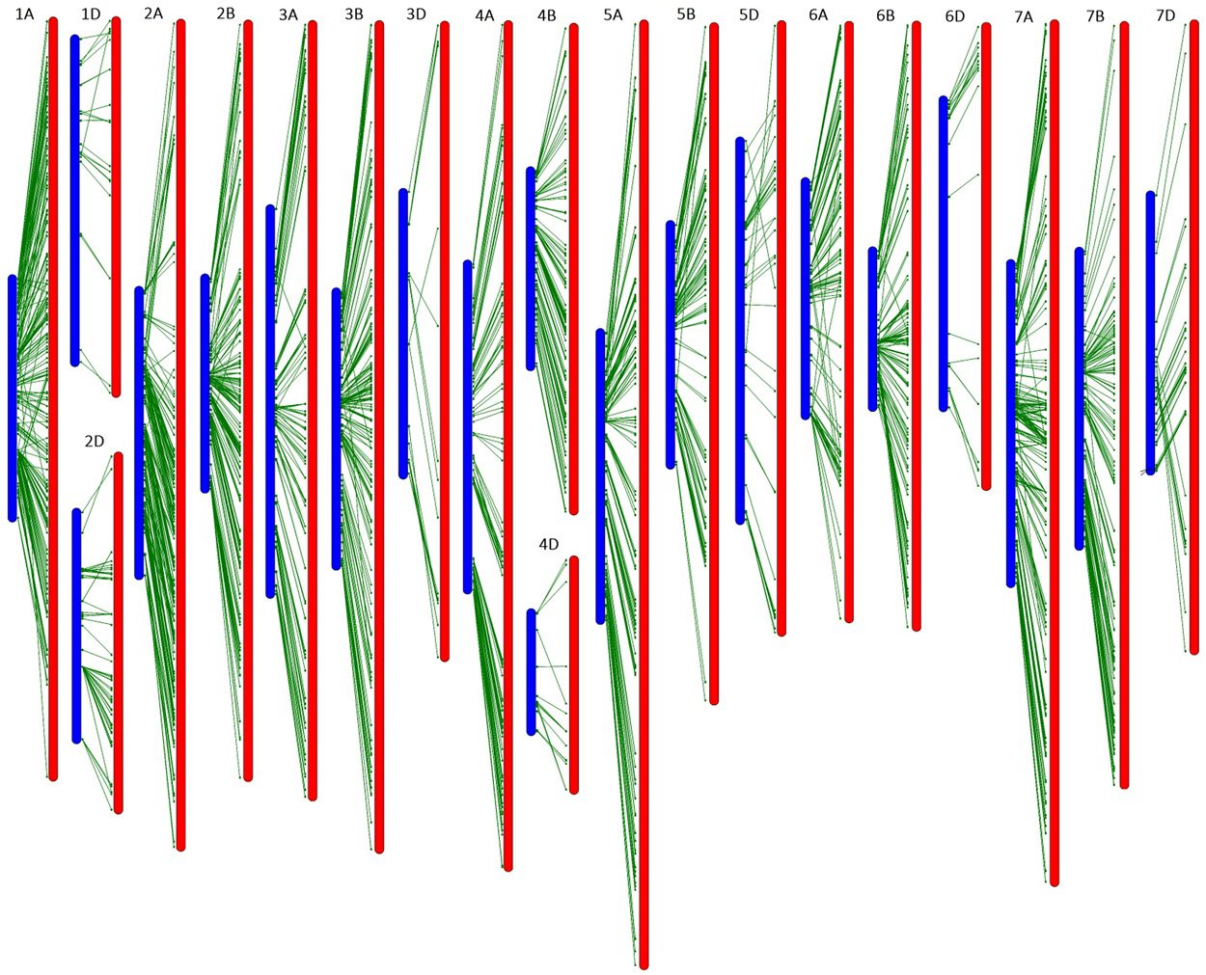
Synteny of the mapped SNPs from the Wheat660K SNP array (in *blue*) with that from the Wheat90K array (in *red*) based on their common CSS-assembled contigs.

**Figure 6:**
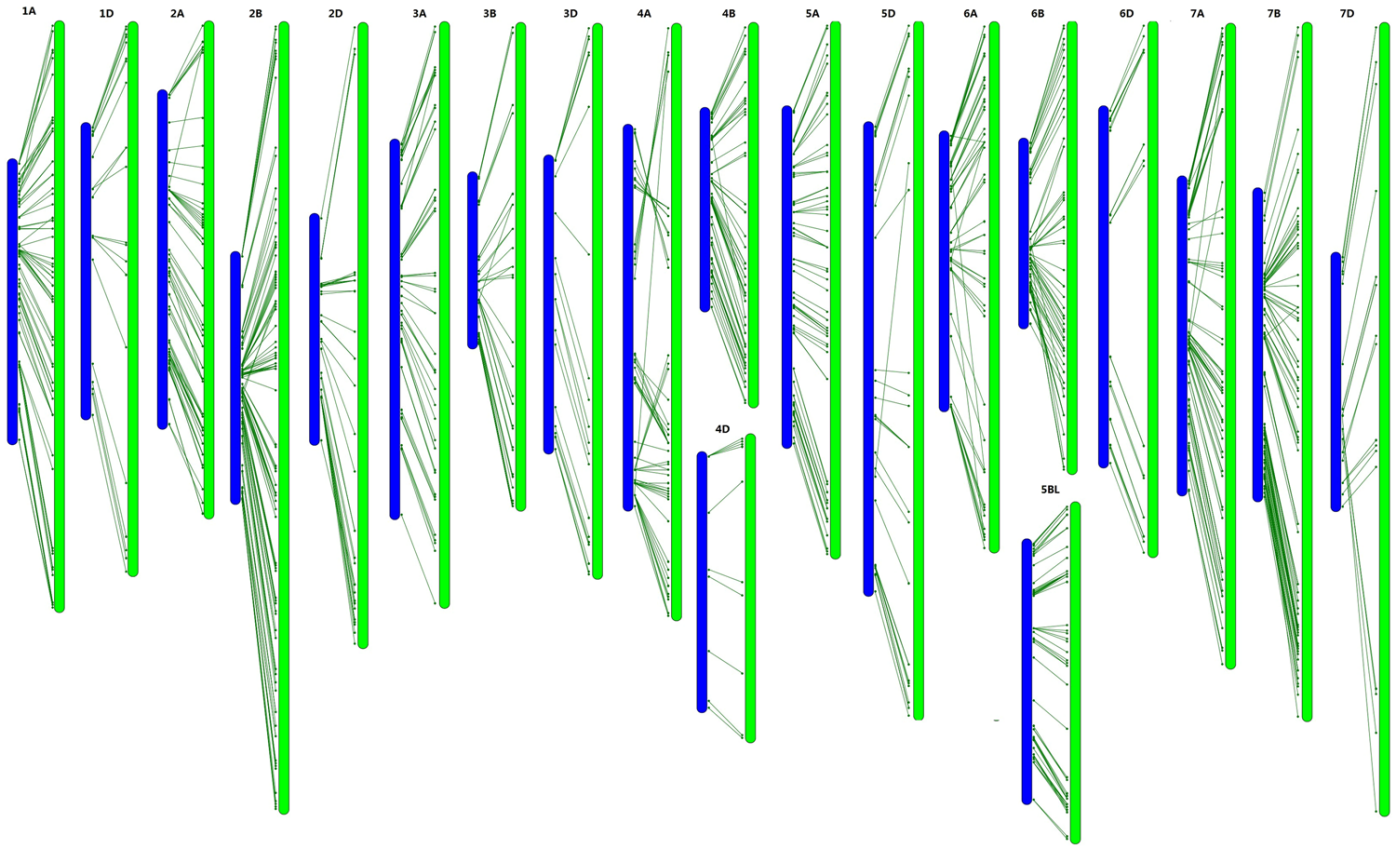
Synteny of the mapped SNPs from the Wheat660K SNP array (in *blue*) with that from the Wheat820K array (in *green*) based on their common CSS-assembled contigs. Notes: The genetic maps of chromosomes 3A, 4B, 5D, 6D and 7B derived from the Wheat820K SNP array are shown in inverted form, with long arms at the top and short arms at the bottom^18^. In this figure, these inversions were corrected.

In previous studies, 7504 SNPs from the Wheat9K SNP array^12^, 46977 SNPs from the Wheat90K array^16^, and 56505 SNPs from the Wheat820K SNP array^18^ were genetically mapped to the hexaploid wheat genome. These SNPs have also been physically assigned to the corresponding CSS-assembled contigs. Based on the common CSS contigs, we analysed the synteny of the mapped SNPs (Wheat660K SNP array vs. Wheat90K array and Wheat660K SNP array vs. Wheat820K SNP array) across different mapping populations (Figs 5 and 6). These common contigs were generally aligned with the chromosomes in a consistent order across different mapping populations, verifying the accuracy and credibility of our high-density genetic map. Regarding the total map length, the Wheat90K/820 SNP consensus integrative genetic map is 2.4/1.9 times longer (data not shown) than that of the present map. The increased genetic map length is proportionate to the increased mapping population size^16, 18^. A relatively small mapping population size resulted in limited identification of recombination events and lower resolution of the genetic map, contributing to the short map length in this study^24, 25^. In addition, comparative analysis among these three SNP maps revealed that genetic maps of chromosomes 3 A, 4B, 5D, 6D, and 7B derived from the wheat 820 K SNP array were inverted, with long arms at the top and short arms at the bottom (data not shown)^18^. Chromosome 4 A, derived from the Wheat820K SNP array, might be involved in chromosomal rearrangement compared with the genetic maps derived from the Wheat90K SNP array and our Wheat660K SNP array. The genetic information from the common contigs and their genetic collinearity analysis across different mapping populations will lay the foundation for obtaining a consensus integrative genetic linkage map.

The SNP order in the present KJ-RIL genetic map was also in good agreement with that in the physical position, with the exception of chromosome 7DL, in which a segment inversion was identified (Fig. 3; Figure S2; Table S3). A previous report showed that wheat chromosome 7 was likely to involve a complex interchange^26^. These findings prompted us to search for candidate genes of targeted major QTLs. In wheat, a majority of recombination events occurred on the most distal portions of the chromosomal arms, whereas the recombination events tend to be suppressed around the centromere^3, 27, 28^. These characteristics result in a low resolution of the genetic map in the centromeric region, which was evident in the small genetic distance in the KJ-RIL genetic map corresponding to a large physical region around the centromere compared with the most distal portions of the chromosomal arms (Fig. 3; Figure S2; Table S3). These findings also indicate the difficulty of high-resolution mapping and map-based cloning of a QTL around the centromere because of the low coverage of genetic markers and the suppression of recombination events.

The genetic and genomic research of wheat has lagged behind similar research regarding other important crops, such as rice and maize^29^. Conservation of gene identity and collinearity among gramineous plants will depend on the rates of genome/gene evolution and rearrangement in the investigated species^22^. There is a high level of genome-synteny among gramineous plants, especially wheat, *Brachypodium* and rice, with wheat being more closely related to *Brachypodium* than to rice^22, 23, 30–32^. Wheat improvement programs can benefit from the use of comparative genetics to transfer information about genes from model species to wheat, to help identify genes controlling traits of interest, and to assess within-species allelic diversity so that the best alleles can be identified and assembled in superior varieties.

In this study, synteny analyses among common wheat, *Brachypodium*, rice, sorghum and maize genomes were performed based on the collinearity of the corresponding orthologous genes (best hits of CDSs) (Table S4; Figures S3 and S4). Features of the wheat-grass genome relationships revealed in this study included a high frequency of breakdown in microcollinearity throughout the genomes compared to the previous RFLP-based maps^22, 31, 33–36^. Both large and especially small synteny blocks across wheat chromosomes and grass family chromosomes were observed in this study. These features might be attributed to the higher number of markers used in this study. More recently, Russo *et al*.^32^ conducted collinearity analysis across durum wheat, *Brachypodium*, and rice based on a high-density durum wheat genetic map derived from the Wheat90K SNP assay. Both large and especially small synteny blocks across wheat chromosomes and grass family chromosomes were also observed in that study. Based on a high-density genetic map, we documented a wheat genome perspective of homologous sorghum and maize genome locations based on comparative sequence analysis. A wheat genome view of homologous gramineous plant genome locations based on comparative sequence analysis would considerably improve the predictability and efficiency of information transfer, and would be benefit evolutionary studies.

Wheat yield is determined by three yield components: productive spikes per unit area, KNPS, and kernel weight, determine wheat yield. Among them, the KNPS value has steadily increased, indicating a substantial contribution of increased KNPS to increased wheat yield^37, 38^. Over the past several decades, numerous QTLs (or genes) for wheat kernel number have been documented based on both linkage-mapping and association-mapping analyses^4, 38–54^. Some of these studies have also reported QTLs for KNPS on chromosome 4 A^4, 40, 44, 50, 51, 53, 54^. Based on genetic marker sequence flanking for these QTLs and the recently released WGS assembly, we compared physical positions with *qKnps-4A* to determine whether they were common interacting QTLs or regions across genetic backgrounds. The results are shown in Supplementary Table S6. *qKnps-4A* from our previous research shared a confidence interval (4 A:664209916–4 A:736771450) with *qKnps-4A* in this study (4 A:680398739–4 A:683638403). Interestingly, *qKnps-4A* has been detected in both the WY and WJ populations (two related RIL populations sharing one common parental line of Weimai 8)^4^. *QKNS.caas-4AL* detected by Gao *et al*.^54^, also shared a confidence interval (4 A:632 864 778–4 A:688 093 018) with *qKnps-4A* in our study. These coincidences confirmed the authenticity of *qKnps-4A*, which should be subjected to fine mapping and map-based cloning in the future. In fact, this work is being conducted based on the secondary mapping population of *qKnps-4A*’s (data not shown).

QTL mapping based on the primary mapping population could precisely characterize and locate genes underlying specific agronomic traits, which was also true for both major and moderate/minor QTLs^55^. Previous studies have confirmed that tagged or cloned genes were near their original QTL positions (logarithm of the odds (LOD) peak)^56–67^. In this study, *qKnps-4A* was repeatedly identified using MapQTL 6.0, IciMapping 4.1, and QTLNetwork 2.0, based on mathematical models of composite interval mapping (CIM), inclusive composite interval mapping (ICIM) and the mixed linear model approach (MLMA), respectively. In addition, the KNPS values of the 188 KJ-RILs and their parental lines were evaluated in 10 different environments. Based on the 10 environmental phenotypic values along with our high-density genetic map, we confirmed QTL peak position with the aforementioned QTL mapping software and found the *Ax-109844107–Ax-110540586* overlapping confidence interval, which spanned 3.23 Mb (4 A:680 398 739*–*4 A:683 638 403) in physical position (Table 1; Fig. 4; Figures S5, S6, S7). In addition, *qKnps-4A* has been mapped to 4 A:664 209 916*–4* *A:*736 771 450 in both the WY and WJ populations^4^ and to 4 A:632 864 778–4 A:688 093 018 in the Zhou 8425B/Chinese Spring population^54^ (Supplementary Table 7). These coincidences across different genetic backgrounds, multiple environments and diverse QTL models strongly supported the hypothesis that the genes underlying *qKnps-4A* are likely located within 4 A:680398739*–*4 A:683638403. One of the 65 predicated genes within this interval might be the candidate gene for *qKnps-4A* (Figure S8). This information is very valuable for future high-resolution mapping and map-based cloning of *qKnps-4A*.

Using *Ax-110540586* as a probe, the 188 KJ-RILs were divided into two groups, one group with alleles from KN9204 and the other with alleles from J411, to perform mean comparisons regarding KNPS. The positive alleles from *qKnps-4A*’s increased the KNPS value from 3.0 to 4.6, indicating a tremendous potential for their application in wheat molecular breeding programmes designed to increase yield (Figure S9). To characterize the use of *qKnps-4A*’s positive alleles in wheat breeding programmes, we dissected the genotypes of 65 KN9204-derived advanced lines/authorized varieties, three parental lines of KN9204, three control varieties from the Winter Wheat Performance Trial of the Northern Huang-Huai Regional Nursery of China, and CS near *Ax-109844107–Ax-110540586* (Figure S10). None of KN9204′s three parental lines carried favourable alleles for increasing KNPS, which accounted for the negative alleles of KN9204 at this QTL. Only 3 (4.6%) of the 65 authorized/advanced lines derived from KN9204 lines carried favourable alleles for increasing KNPS. Interestingly, two advanced lines (KN1002-13-10 and O-97) carried heterozygous alleles, which were used to develop the secondary mapping population of *Knps-4A* via self-cross (data not shown). Of the three control varieties from the Winter Wheat Performance Trial of the Northern Huang-Huai Regional Nursery of China, S4185 carried negative alleles, decreasing KNPS; chromosomal regions of *Knps-4A* in LX99 and JM22 were recombinant regions with alleles that cannot be categorized as favourable or negative alleles that increase or decrease KNPS; and CS carried favourable alleles, increasing KNPS. These findings indicated that favourable *qKnps-4A* alleles have not been fully utilized in wheat breeding programmes in the Huang-Huai winter wheat region in China. Wheat breeders should strengthen the selection of *qKnps-4A* favourable alleles in molecular breeding programmes aimed at the development of high-yield varieties.

In summary, this paper reports a high-density wheat genetic map based on an individual mapping population. In total, 119 001 SNP markers derived from the Wheat660K SNP array were mapped onto the KJ-RIL genetic map. We observed good good collinearity of our high-density genetic map with the Wheat90K and Wheat820K consensus genetic maps, increasing the possibility of obtaining a consensus integrative higher-density wheat genetic map in the future. This high-density genetic map is also in good accordance with the recently released wheat genome assembly. Our high-density wheat genetic map provides a major resource for future wheat genetic and genomic research. Moreover, this paper provides an overview of the structural relationships between wheat and other gramineous plant genomes based on comparative genomics analysis. Finally, a major stable QTL for kernel number was thoroughly characterized based on this high-density genetic map and comparative genomics analysis.

## Experimental procedures

### Plant material

In this study, F_8:9_ KJ-RILs derived from a cross between KN9204 and J411 were used for map construction and QTL analysis. The original KJ-RIL population contained 427 RILs. In total, 188 randomly sampled lines from the 427 KJ-RILs were used for genetic linkage analysis. In addition, 65 KN9204-derived advanced lines/authorized varieties, three parental lines of KN9204, three control varieties from the Winter Wheat Performance Trial of the Northern Huang-Huai Regional Nursery of China, and CS (Table S7) were genotyped to trace the key chromosomal segment harbouring the major stable QTL for kernel number per spike (KNPS).

### Phenotyping

KNPS values of the 188 KJ-RILs and their parental lines were evaluated in ten different environments (five trials that included both low- and high-nitrogen treatments). The nitrogen treatments, field arrangements and experimental designs of the ten environments were performed as described previously^3, 68, 69^.

### Genotyping

For all subjects, leaf tissues were sampled. Genomic DNA was extracted and hybridized on the Wheat660K SNP genotyping array by Compass Biotechnology Company (Beijing, China). The DNA samples were prepared, and the chip genotyping was performed on the Wheat660K SNP array according to the Affymetrix Axiom 2.0 Assay Manual Workflow protocol. DNA integrity was confirmed on agarose gels, and DNA quantity was measured spectrophotometrically. The Wheat660K chip contains 630517 markers (http://wheat.pw.usda.gov/GG2/index.shtml). Variant quality from the Wheat660K chip genotyping was initially assessed according to Affymetrix best practices. The 188 RILs and their parents were aslo assayed using the ‘Wheat *PstI* (*TaqI*) 2.3D’ DArT array (the medium density array) (http://www.triticarte.com.au/). The PCR-based markers were genotyped as described in our previous study^3^.

### Genetic map construction

The 188 KJ-RILs and their parental lines were genotyped with the Wheat660K SNP array. SNPs were rejected if they showed minor allele frequency (defined as frequency <0.3) or contained >10% missing data. Markers were binned based on their segregation patterns in the KJ-RIL population using the BIN function in IciMapping 4.1 (http://www.isbreeding.net/) according to Winfield *et al*.^18^. Markers that shared their segregation pattern with at least one other marker were retained. One marker was chosen to represent each bin on the basis of the least amount of missing data or, when the percentage of missing data was equal, at random. Markers were tested for significant segregation distortion using a chi-square test. SNPs were sorted into groups using the MAP function in IciMapping 4.1, with the previously mapped 591 loci serving as anchored markers^3^. A logarithm of the odds (LOD) score of 3.5 and a recombination fraction of 0.3 were used to sort the SNPs with the Kosambi mapping function^70^. Groups were ordered with the Kosambi mapping function within the JoinMap v. 4.0, using a LOD score ≥3 after preliminary analysis of SNPs with LOD scores ranging from 2 to 10. The long and short arms of each chromosome were identified from the IWGSC wheat survey sequence (http://www.wheatgenome.org/), and groups were orientated to have the short arm above the long arm. MapChart 2.2 (http://www.biometris.nl/uk/Software/MapChart/) was used to draw the genetic map.

### QTL mapping analysis

The QTLs for KNPS were identified using MapQTL 6.0 (based on CIM, https://www.kyazma.nl/index.php/mc.MapQTL/), IciMapping 4.1 (based on ICIM, http://www.isbreeding.net/software/?type=detail&id=18), and QTLNetwork 2.0 (based on MLMA, http://ibi.zju.edu.cn/software/qtlnetwork/). For MapQTL 6.0 and IciMapping 4.1, the average KNPS values of each KJ-RIL in each environment were used for individual environment QTL mapping analysis. For QTLNetwork 2.0, the KNPS values of each KJ-RIL in each replication of the 10 environments were assembled to perform combined QTL analysis across environments to identify QTLs with additive-by-environment (A by E) interaction effects. The overlapping confidence intervals detected with the abovementioned programs were used to predict the candidate genes based on the sequence information of the flanking markers and the IWGSC WGA v0.4 assembly of chromosome 4A (https://urgi.versailles.inra.fr/download/iwgsc/IWGSC-WGA_Sequences/).

### Comparative genomic analysis

The SNP flanking sequences mapped in the KJ-RIL map were kindly provided by Professor Jia JZ. We used the Basic Local Alignment Search Tool (BLAST) (ftp://ftp.ncbi.nlm.nih.gov/blast/executables/release/) to align the SNP probes to the IWGSC survey sequences (contigs). All IWGSC survey sequences were downloaded from http://www.wheatgenome.org/. In addition, contig sequences to which the SNPs were best hits were screened in a BLASTN search against the coding sequences (CDSs) of *B. distachyon*, rice (*O. sativa* L.), maize (*Z. mays* L.), and sorghum (*S. vulgare* L.). All CDSs were downloaded from http://plants.ensembl.org/index.html. An expectation value (*E*) of 1E^*−*10^ was used as the significance threshold. Synteny analyses with common wheat, *Brachypodium*, rice, maize and sorghum genomes were performed based on the SNP orders in the KJ genetic map and on the corresponding CDSs in the genome sequences of *Brachypodium*, rice, maize and sorghum genomes.

Using the sequences of the markers (including SNPs, PCR-based markers and DArTs), we conducted comparative genomics analysis against the contigs assembled in the chromosome survey sequencing (CSS) project. All of the contig sequences were downloaded from https://wheat-urgi.versailles.inra.fr/Seq-Repository. More recently, the genome assembly of *T. aestivum* cv. Chinese Spring (CS) has been released (https://urgi.versailles.inra.fr/download/iwgsc/IWGSC-WGA_Sequences/). Based on SNP flanking sequences, we assigned SNPs to this wheat genome assembly.

## Electronic supplementary material

Table S2

Table S3

Table S4

Table S5

Supplementary information
