# Supplementary material for: Utilization of a Wheat660K SNP array-derived high-density genetic map for high-resolution mapping of a major QTL for kernel number
Source: Sci Rep. 2017 Jun 19;7:3788. doi: 10.1038/s41598-017-04028-6 (PMC5476560; doi:10.1038/s41598-017-04028-6)
Supplement: Supplementary file 5 — Supplementary information [file 41598_2017_4028_MOESM5_ESM.doc]

**Utilization of a Wheat660K SNP array-derived high-density genetic map** **for high-resolution mapping of a major QTL for kernel number**

**Fa Cui^1,2,7^,*, Na Zhang^1,6,+^, Xiao-li Fan^1,3,+^, Wei Zhang^1,7^,*, Chun-hua Zhao^2^, Li-juan Yang^4^, Rui-qing Pan^1,6^, Mei Chen^1,6^, Jie Han^1,6^, Xue-qiang Zhao^7^, Jun Ji^1,7^, Yi-ping Tong^7^, Hong-xia Zhang^2^, Ji-zeng Jia^5^, Guang-yao Zhao^5^,* and Jun-ming Li^1,7^,***

^1^Center for Agricultural Resources Research, Institute of Genetics and Developmental Biology, Chinese Academy of Sciences, Shijiazhuang, 050022, China

^2^Genetic Improvement Centre of Agricultural and Forest Crops, College of Agriculture, Ludong Unversity, Yan’tai, 264025, China

^3^Chengdu Institute of Biology, Chinese Academy of Sciences, Chengdu, 610041, China

^4^ Xinxiang Academy of Agricultural Sciences, Xinxiang, 453000, China

^5^Institute of Crop Science, Chinese Academy of Agricultural Sciences, Beijing, 100081, China

^6^ University of Chinese Academy of Sciences, Beijing, 10049, China

^7^State Key Laboratory of Plant Cell and Chromosomal Engineering, Chinese Academy of Sciences, Beijing, 100101, China

**^*^Correspondence:** [sdaucf@126.com](mailto:sdaucf@126.com,); edithor@126.com; [ljm@sjziam.ac.cn](mailto:ljm@sjziam.ac.cn); zhaoguangyao@caas.cn

**^+^** These authors contributed equally to this work.


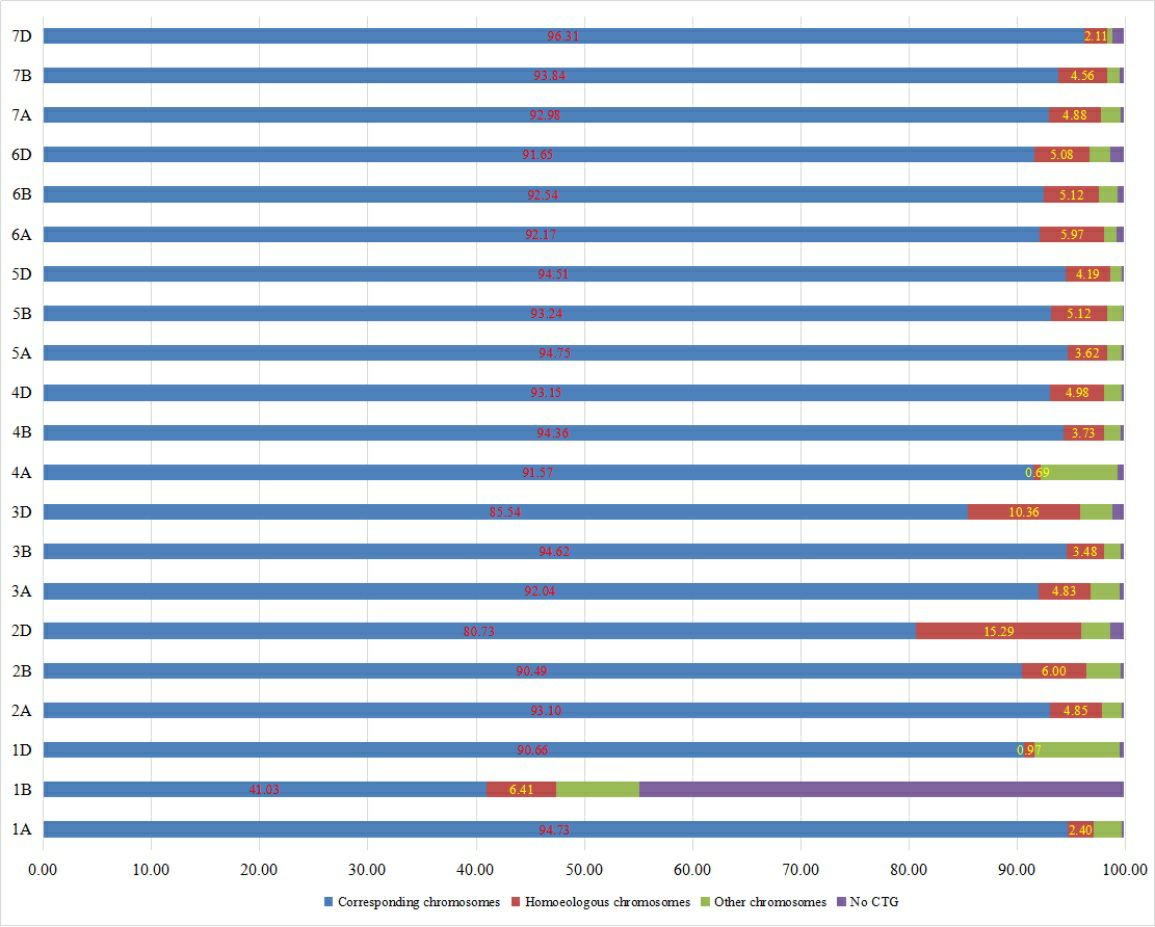


**Figure S1** **Comparison of the genetic and physical positions of the mapped markers.** The length of the histogram in *blue* indicates the percentage of markers with coincident physical and genetic positions (identical chromosome); *red* indicates the percentage of markers that were mapped to homoeologous chromosomes compared with the physical positions; *green* indicates the percentage of markers with inconsistent physical and genetic positions (in disorder); and *purple* indicates the percentage of markers with unknown physical positions. The physical positions are from the Chinese Spring contigs which were the best hits for the corresponding markers.

**Figure S2** Comparison of collinearity between markers in the wheat genetic and physical maps. The 21 wheat chromosomal genetic maps were referenced to the KJ-RIL-derived high-density genetic map. The 21 wheat chromosomal physical maps were constructed based on assigning 116 261 SNPs to the [*Triticum* *aestivum*](http://plants.ensembl.org/Triticum_aestivum) cv. Chinese Spring genome assembly using SNP flanking sequences as the query.


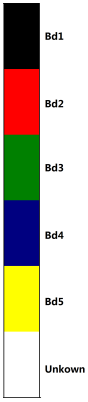


**Figure S3a** KJ-RIL wheat genetic map view showing the *B.* *distachyon* chromosomal locations for the most similar *Brachypodium* coding sequences (CDSs). Each coloured box represents a Brachypodium CDSs match at an expectation value (*E*) of 1e–10 and identity >95% (see key, bottom right).


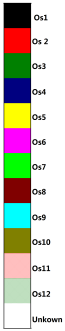


**Figure S3b** KJ-RIL wheat genetic map view showing the rice (*Oryza sativa* L.) chromosomal locations for the most similar rice coding sequences (CDSs). Each coloured box represents a rice CDS match at an expectation value (*E*) of 1e–10 and identity >95% (see key, bottom right).


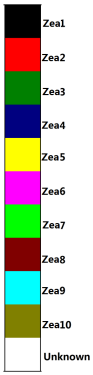


**Figure S3c** KJ-RIL wheat genetic map view showing the maize (*Zea mays* L.) chromosomal locations for the most similar maize coding sequences (CDSs). Each coloured box represents a maize CDS match at an expectation value (*E*) of 1e–10 and identity >95% (see key, bottom right).


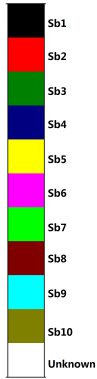


**Figure S3d** KJ-RIL wheat genetic map view showing the Sorghum (*Sorghum vulgare* L.) chromosomal location for the most similar Sorghum coding sequences (CDSs). Each coloured box represents a Sorghum CDS match at an expectation value (*E*) of 1e–10 and identity >95% (see key, bottom right).


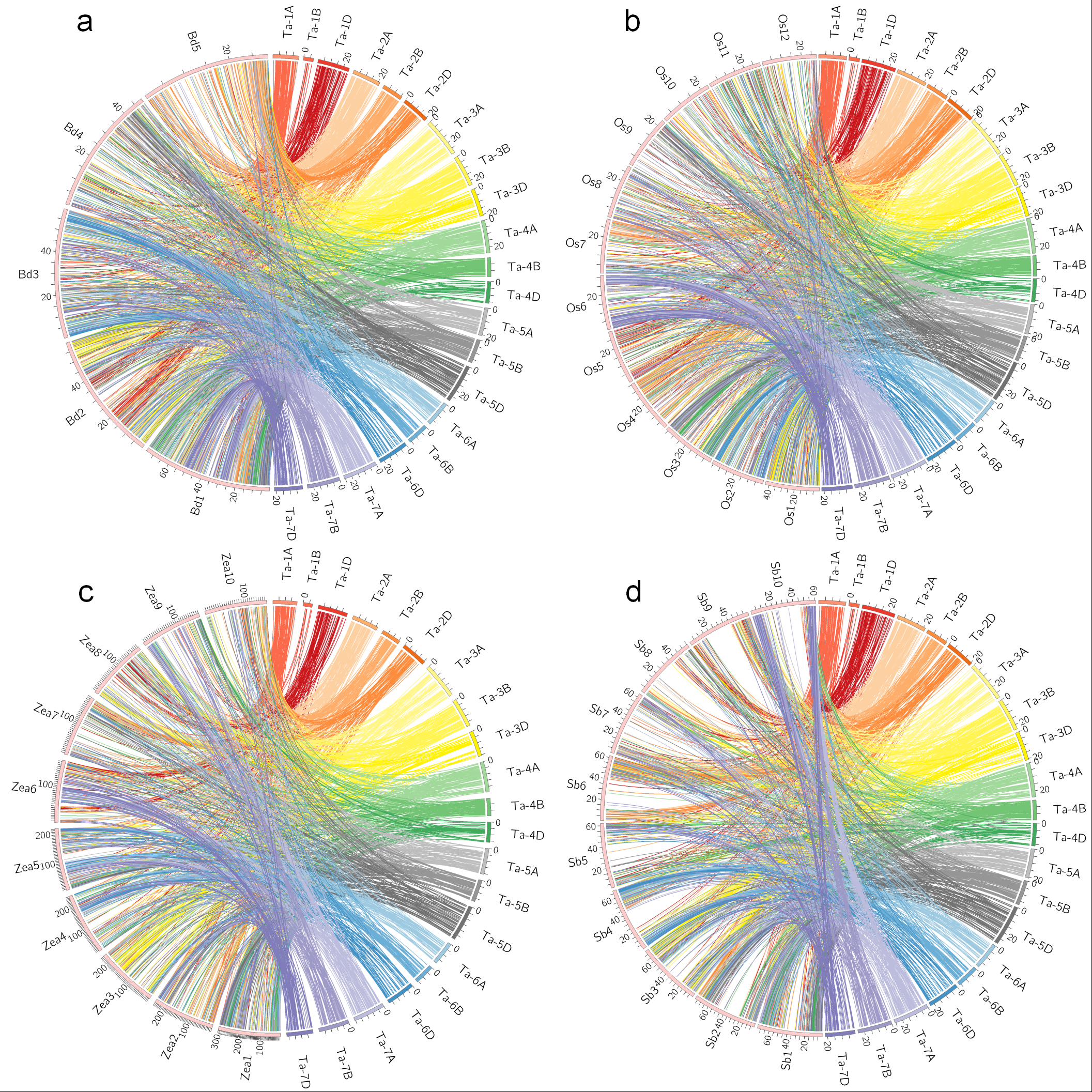


**Figure S4** Shared synteny between wheat chromosomes and the chromosomes in *Brachypodium*, rice, maize and sorghum. Wheat chromosomes are indicated on the right side of the circle, and each colour corresponds to a chromosome. The chromosomes of *B.* *distachyon* (Bd1-5) (a), rice (Os1-12) (b), Maize (Zea1-Zea10) (c) and sorghum (Sb1-10) (d) are represented on the left side of the circle. The lines represent the relationships between mapped genes on the wheat chromosomes and the orthologous genes in *B. distachyon*, rice, sorghum, and maize. The order of wheat genes in a given deletion bin is predicted by the alignment of contigs along the KJ-RIL map.

**Notes:** This figure was trimmed to eliminate redundant information where more than one mapped wheat SNP matched the same *B*. *distachyon*/ rice/maize/sorghum CDS region without providing additional information regarding wheat chromosome location.


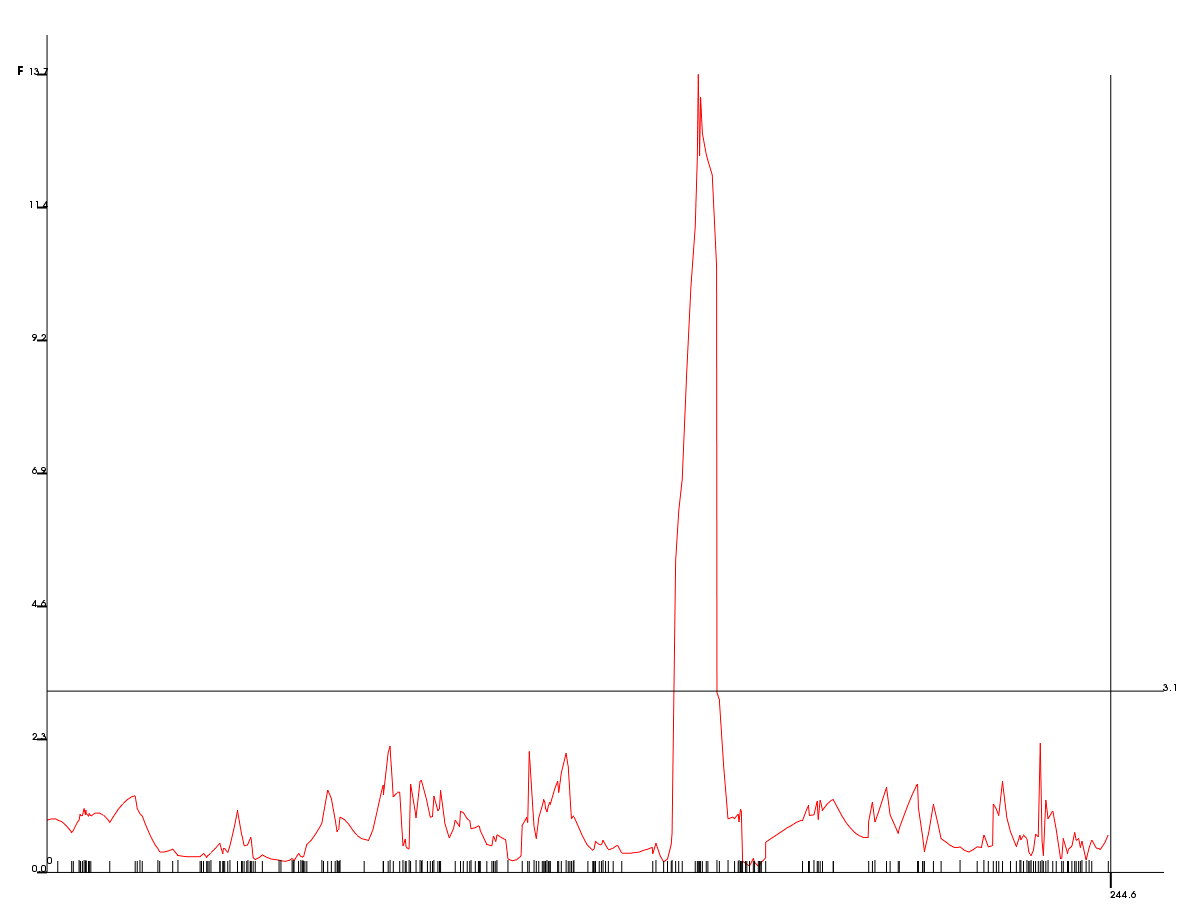


**Figure S5** *F* values of the QTLs for kernel number per spike (KNPS) based on QTLNetwork 2.0 on chromosome 4A in the 10 combined environments


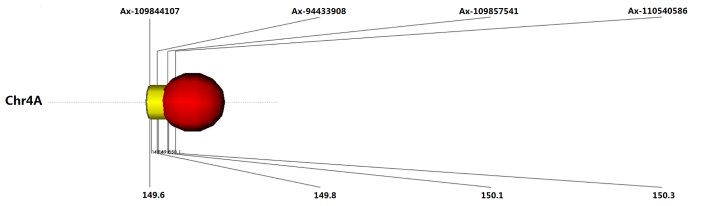


**Figure S6** The position of *qKnps-4A* as detected by QTLNetwork 2.0 in the 10 combined environments.

**Figure S7** Comparison of collinearity between marker in the wheat genetic and physical maps at *qKnps-4A* (149.1 cM–152.0 cM in genetic position vs. 4A:677618526–4A:684269262 in physical position).

**Figure S8** Predicted genes of *qKnps-4A* in 3.23 Mb of 4A:680398739–4A:683638403 in [*Triticum* *aestivum*](http://plants.ensembl.org/Triticum_aestivum).


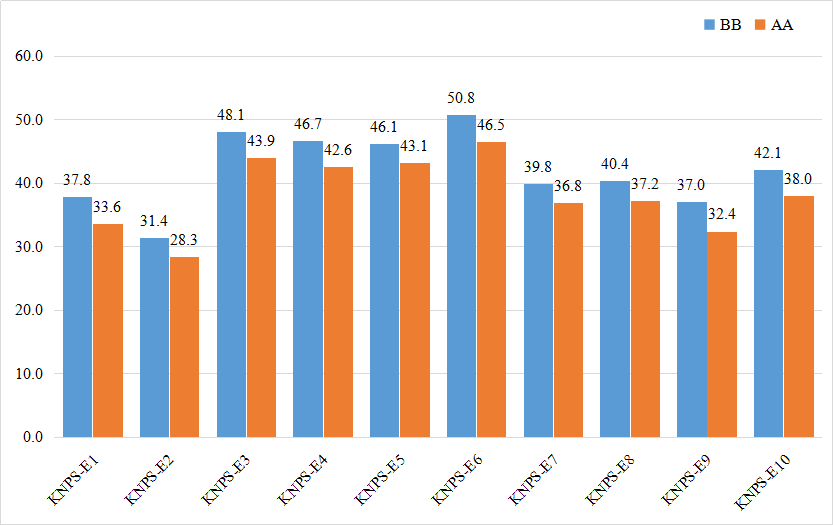


**Figure S9** Mean comparison of the kernels per spike in the two groups of the 188 KJ-RILs using *Ax-110540586* as a probe in the ten different environments.


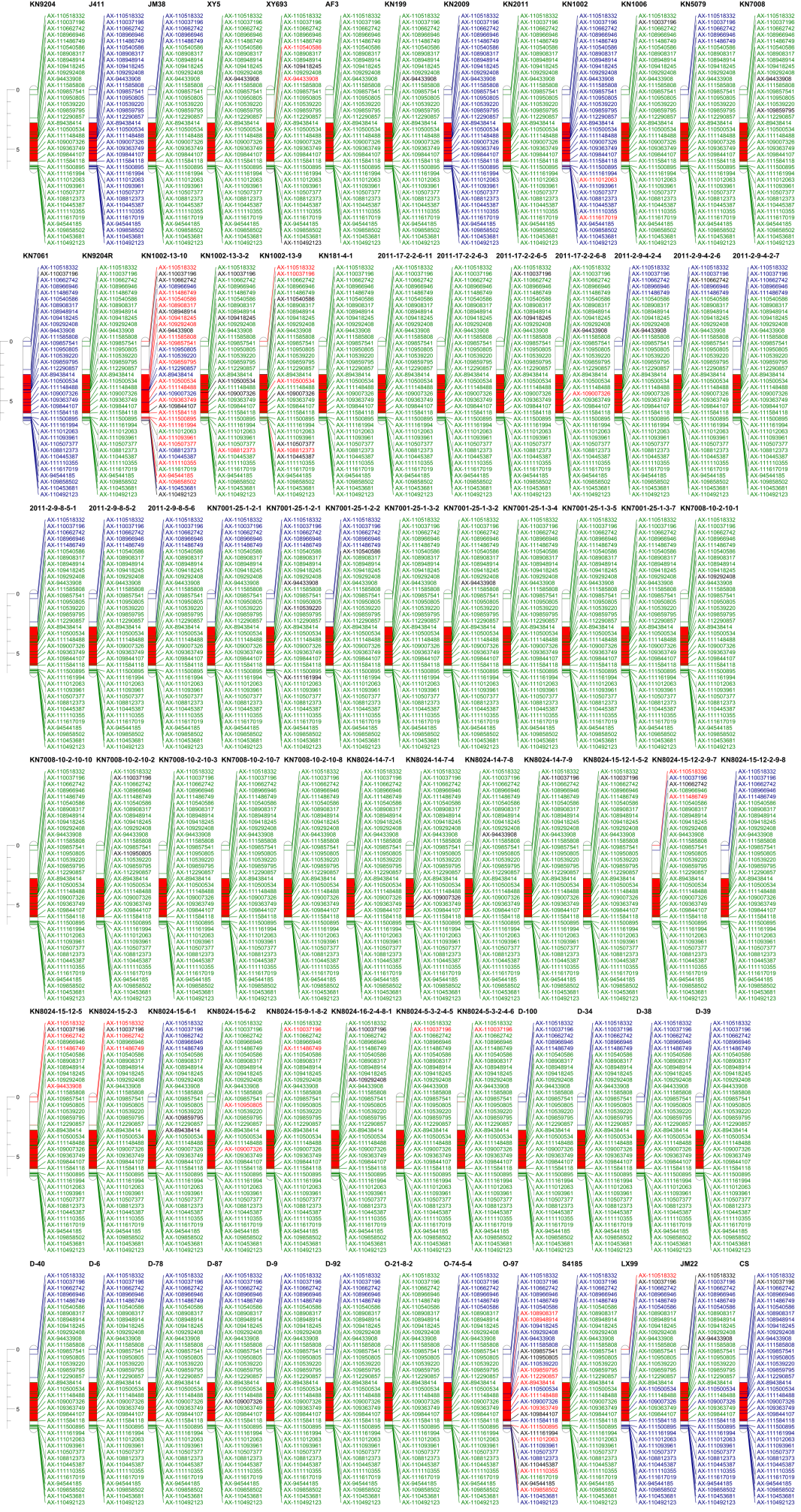


**Figure S10** Genotypes of 67 KN9204-derived advanced lines/authorized varieties, three parental lines of KN9204, three control varieties from Winter Wheat Performance Trial of the Northen Huang-Huai Regional Nursery of China and CS near *Ax-109844107–Ax-110540586.*

**Table S1** SNP categories in the 265 accessions.

| SNP category | Count | Percentage (%) |
| --- | --- | --- |
| PHR | 188,040 | 29.8 |
| NMH | 133,246 | 21.1 |
| OTV | 18,471 | 3.0 |
| MHR | 163,308 | 25.9 |
| CRBT | 22,635 | 3.6 |
| Other | 91,425 | 14.5 |
| HomHomResolution | 13,392 | 2.1 |
| Total | 630,517 | 100.0 |

(i) PHR, which were codominant and polymorphic, with at least two examples of the minor allele; (ii) NMH, which were polymorphic and dominant, with two clusters observed; (iii) OTV, which had four clusters, one representing a null allele; (iv) MHR, which were monomorphic; (v) CRBT, where the SNP call rate was below threshold, but other cluster properties were above threshold; and (vi) Other, where one or more cluster properties were below threshold.

**Table S6:** Comparisons of *qKnps-4A* with previous studies for sensitivity across different genetic backgrounds

| Flanking markers | Physical position (bp) | Reference |
| --- | --- | --- |
| *Xwg622*–*Xmwg549*–*Xabg390* | 4A:600913033–636315952 | Börner et al. (2002) |
| *Xwmc48*–*Xwmc89*–*Xwmc420* | 4A:515847927–4A:538217339 | Kirigwi et al. (2007) |
| *Xwmc468*–*Xbarc170*  *Xbarc105*–*Xgdm61*  *Xbarc301*–*Xbarc122*  *Xbarc59*–*Xsrap7* | 4A:607887551–4A:617000592  4A:565760575  –  4A:640991260 | Tang et al. (2011) |
| *Xbarc170*–*Xwmc707*–*Xwmc258*  *Xbarc1047*–*Xwmc718* | 4A:619914714  4A:629116694 | Wang et al. (2011) |
| *Xwmc497*–*Xwmc313*–*Xgwm160*–*Xwmc232* | 4A:700738965–4A:739515211 | Patil et al. (2013) |
| ***wPt-0610***–***wPt-4487***–***wPt-5172***–***wPt-5003***–***Xwmc468*** | **4A:664209916**–4A:**736771450** | Cui et al. (2014b) |
| ***wsnp_Ra_rep_c70233_67968353***–***Kukri_c20012_1362*** | **4A:632864778–4A:688093018** | Gao et al. (2015) |
| ***Ax-109844107–Ax-110540586*** | **4A:680398739–4A:683638403** | The present study |

**References**

Börner, A., Schumann, E., Fürste, A., Cöster, H., Leithold, B., Röder, M. and Weber, W. (2002) Mapping of quantitative trait loci determining agronomic important characters in hexaploid wheat (*Triticum aestivum* L.). *Theor. Appl. Genet.* **105**, 921–936.

Kirigwi, F.M., Van Ginkel, M., Brown-Guedira, G., Gill, B.S., Paulsen, G.M. and Fritz, A.K. (2007) Markers associated with a QTL for grain yield in wheat under drought. *Mol*. *Breeding*, **20**, 401–413.

Tang, Y.L., Li, J., Wu, Y.Q., Wei, H.T., Li, C.S., Yang, W.Y. and Chen, F. (2011) Identification of QTL for yield-related traits in the recombinant inbred line population derived from the cross between a synthetic hexaploid wheat- derived variety Chuanmai 42 and a Chinese elite variety Chuannong 16. *Agric. Sci . China,* **10**, 1665–1680.

Wang, J., Liu, W., Wang, H., Li, L., Wu, J., Yang, X., Li, X. and Gao, A. (2011) QTL mapping of yield-related traits in the wheat germplasm 3228. *Euphytica*, **177**, 277–292.

Patil, R.M., Tamhankar, S.A., Oak, M.D., Raut, A.L., Honrao, B.K., Rao, V.S. and Misra, S.C. (2013) Mapping of QTL for agronomic traits and kernel characters in durum wheat (*Triticum durum* Desf.). *Euphytica*, **190**, 117–129.

Cui, F., Zhao, C., Ding, A., Li, J., Wang, L., Li, X., Bao, Y., et al. (2014b) Construction of an integrative linkage map and QTL mapping of grain yield-related traits using three related wheat RIL populations. *Theor. Appl. Genet.* **127**, 659–675.

Gao, F.M., Wei, W.E., Liu, J.D., Rasheed, A., Yin, G.H., Xia, X.C., Wu, X.X. and He, Z.H. (2015) Genome-wide linkage mapping of QTL for yield components, plant height and yield-related physiological traits in the Chinese wheat cross Zhou 8425B/Chinese Spring. *Front. Plant Sci*. **6**,1099. doi: 10.3389/fpls.2015.01099.

**Table S7** Derivatives of the Kenong 9204 pedigree, Kenong 9204’s parental lines, three control varieties from Winter Wheat Performance Trial of the Northen Huang-Huai Regional Nursery of China, and Chinese Spring accessions used in this study

| Line | Abbrevation | Generation | NO. of lines | Pedigree |
| --- | --- | --- | --- | --- |
| Kenong 2011 | KN2011 | Authorized variety (F_13_ ) | 11 | Kenong9204/ PZW-9 |
| Kenong 5079 | KN5079 | F_10_ | 1 | Kenong9204/Shi4185//Kenong9204/9331 |
| Kenong 7001 | KN7001 | F_8_ | 8 | Kenong9204/Xinong9848 |
| Kenong 7008 | KN7008 | F_8_ | 7 | Kenong9204/9331//Kenong9204/PZW-9 |
| Kenong 8024 | KN8024 | F_7_ | 15 | Kenong9204/PZW-9//Shannong16 |
| Kenong1002 | KN1002 | F_5_ | 4 | Kenong9204/PZW-9//Jinfeng5027 |
| Kenong 1006 | KN1006 | Authorized variety (F_14_ ) | 1 | Kenong9204/Gaomai5// Kenong9204R |
| Kenong 199 | KN199 | Authorized variety (F_17_ ) | 1 | Kenong9204/Shi4185 |
| Kenong 9204R | KN9204R | F_19_ | 1 | Kenong 9204/148// Kenong 9204///Kenong 9204 |
| Kenong 2009 | KN2009 | Authorized variety (F_13_ ) | 1 | 148/BE-1//148/// Kenong9204/Gao8901////Kenong1095 |
| Kenong 7061 | KN7061 | F_8_ | 1 | Kenong9204/PZW-9//04Zhong38 |
| 181-4-1 | 181-4-1 | F_20_ | 1 | Kenong9204/9331 |
| O-21-8-2 | O-21-8-2 | F_17_ | 1 | Kenong 9204/Gao8901// Z-4-2 |
| O-74-5-4 | O-74-5-4 | F_17_ | 1 | fZ-5-2/148-2 |
| O-97 | O-97 | F_17_ | 1 |  |
| D-6 | D-6 | BC_3_F_3_ | 1 | Ae9349-1/Kn199//Kn199////Kn199/////Kn199 |
| D-9 | D-9 | BC_3_F_3_ | 1 | Ae9349-1/Kn199//Kn199////Kn199/////Kn199 |
| D-34 | D-34 | BC_3_F_3_ | 1 | Ae9351-1/Kn199//Kn199////Kn199/////Kn199 |
| D-38 | D-38 | BC_3_F_3_ | 1 | Ae9351-1/Kn199//Kn199////Kn199/////Kn199 |
| D-39 | D-39 | BC_3_F_3_ | 1 | Ae9351-1/Kn199//Kn199////Kn199/////Kn199 |
| D-40 | D-40 | BC_3_F_3_ | 1 | Ae9351-1/Kn199//Kn199////Kn199/////Kn199 |
| D-78 | D-78 | BC_3_F_3_ | 1 | Ae9351-1/Kn199//Kn199////Kn199/////Kn199 |
| D-87 | D-87 | BC_3_F_3_ | 1 | Ae9351-1/Kn199//Kn199////Kn199/////Kn199 |
| D-92 | D-92 | BC_3_F_3_ | 1 | Ae9351-1/Kn199//Kn199////Kn199/////Kn199 |
| D-100 | D-100 | BC_3_F_3_ | 1 | Ae9355-1/Kn199//Kn199////Kn199/////Kn199 |
| Xiaoyan 5 | XY5 | Authorized variety | 1 | Parental lines of KN9204 |
| Xiaoyan 693 | XY693 | Authorized variety | 1 | Parental lines of KN9204 |
| Aifeng 3 | AF3 | Authorized variety | 1 | Parental lines of KN9204 |
| Jimai 38 | JM38 | Authorized variety | 1 | Control varieties of regional test of Huang-huai winter wheat region in China |
| Jimai 22 | JM22 | Authorized variety | 1 | Control varieties of regional test of Huang-huai winter wheat region in China |
| Liangxing 99 | LX99 | Authorized variety | 1 | Control varieties of regional test of Huang-huai winter wheat region in China |
| Chinese Spring | CS |  | 1 |  |
